# Clinical relevance of miR-423-5p levels in chronic obstructive pulmonary disease patients

**DOI:** 10.1016/j.clinsp.2022.100102

**Published:** 2022-09-23

**Authors:** Xin Zhang, Qing Shi, Lu Xiong, Shiye Shi, Yong Li, Yanhuan Wang, Mingchuan Zhang

**Affiliations:** a Respiratory Department, ChongQing TongLiang People's Hospital, ChongQing, China; b Emergency Department, ChongQing TongLiang People's Hospital, ChongQing, China

**Keywords:** COPD, miR-423-5p, GEO, Receiver operating curve

## Abstract

**Objective:**

This study aimed to examine changes in miRNAs expression profile of COPD patients.

**Methods:**

Thirty-six COPD patients as well as thirty-three healthy volunteers were recruited. Total RNAs were collected from the plasma of each participant. The differentially expressed miRNAs in COPD were screened from the GEO database. RT-qPCR was carried out to detect miRNA expression.

**Results:**

In total, 9 out of 55 miRNAs were expressed differentially in COPD patients. Confirmed by RT-qPCR validation, 6 miRNAs increased while 3 miRNAs decreased. Further analysis of miR-423-5p, which has not been reported in COPD, showed that AUC for the diagnosis of COPD was 0.9651, and miR-423-5p levels was inversely correlated with the duration of smoking.

**Conclusion:**

The present study demonstrates that miR-423-5p is a potential marker for identifying COPD patients.

## Highlights

• Relationship of plasma miR-423-5p expression in COPD patients as well as smoking history.
• 9 miRNAs were dysregulated in COPD patients.
• miR-423-5p has potential value as a clinical diagnosis of COPD.

## Introduction

Chronic Obstructive Pulmonary Disease (COPD), a common respiratory disease, is characterized by continuous respiratory inflammation as well as airflow restriction, and airflow restriction mostly presents an irreversible progressive development.^1^^,^^2^ It is reported that the prevalence of COPD in adults over 40 years of age is 5‒19% globally,^3^ causing a huge social burden. COPD is usually caused by long-term exposure to harmful gases or small particles and is also associated with genes, airway hyperresponsiveness, and pulmonary dysplasia.^4^ Smoking is currently considered to be the most important pathogenic factor for COPD.^5^ In addition, genetic factors such as age growth,^6^ gender difference,^7^ and α-1 antitrypsin deficiency^8^ are the causes of COPD as well.

The pathogenesis of COPD is complex and has not yet been fully clarified. Previously, the pathogenesis of COPD included inflammatory response,^9^ oxidative stress,^10^ protease imbalance,^11^ etc. In recent years, hypotheses such as apoptosis,^12^ respiratory microbial disorder,^13^ and ineffective repair of damaged stem cells^14^ have further improved the research on the pathogenesis of COPD. At present, bronchiectasis drugs such as β2 receptor agonist^15^ and muscarinic antagonist^16^ are mainly used in the clinical treatment of COPD. Anti-inflammatory drugs and antioxidant drugs are also effective treatment methods. Considering the high morbidity and mortality, further study of COPD is necessary.

MicroRNA (miRNA) are short-chain non-coding RNA molecules composed of about 22 nucleotides, which can specifically bind to the target mRNA to inhibit its translation or mediate its degradation, so as to realize the gene regulation at the post-transcriptional level.^17^ It is estimated that miRNA regulates about 25% of all human genes.^18^ Therefore, miRNA involves a large number of different biological processes, such as cell proliferation and differentiation, aging, metabolism as well as inflammation,^19^, ^20^, ^21^ thus miRNA plays a huge role in organisms. Studies have reported that there is a significant imbalance in miRNA in COPD patients.^22^^,^^23^ Further studies have shown that miRNA can affect lung development,^24^ and mediate the generation of inflammation,^25^ thus affecting the occurrence and development of COPD. MiR-423 is a relatively conserved miRNA in humans, mice, pigs, cows, and other species. It can form two mature sequences: miR-423-3p and miR-423-5p. MiR-423 is closely related to many diseases. For example, miR-423-5p can be used as a molecular marker to reflect the severity of liver failure.^21^ MiR-423-3p promotes cell proliferation, migration, and invasion in endometrial cancer,^1^ liver cancer,^13^ gastric cancer,^22^ colorectal cancer^23^ and other cell lines and animal models. However, the role of miR-423-3p in COPD remains unclear.

Therefore, the secondary objective of this study was to select the profiled plasma miRNAs in COPD patients. The primary objective was to explore the relationship between miR-423-5p and COPD and provide potential targets for COPD treatment.

## Materials and methods

### Patients

Samples were gathered from COPD patients (*n* = 36) and healthy volunteers (*n* = 33) at ChongQing TongLiang people's Hospital. The healthy individuals were not smokers. In the morning, 5 mL of median cubital venous blood was collected on an empty stomach, centrifuged at -4°C and 2000 r/min for 10 min. Then the serum was separated and stored in a -70°C refrigerator for further experiments.

The study was approved by ChongQing TongLiang people's Hospital (Ethical number 2020‒33). Signed informed consent forms were obtained from each individual.

### RNA isolation

Isolation of RNA from patients’ plasma (200 µL) was performed using a miRNeasy Serum/Plasma Advanced Kit (Qiagen). Each miRNA sample had a total volume of 30 µL and was stored at -80°C prior to cDNA synthesis.

### Real-time quantitative polymerase chain reaction (RT-qPCR)

Total RNA was extracted by the TRIzol® reagent (Invitrogen; Thermo Fisher Scientific, Inc.). Reverse transcription and qPCR were performed by BlazeTaq One-Step SYBR Green RT-qPCR Kit (with ROX) (QP071; GeneCopoeia, Inc., USA) on a SEDI Thermo Cycler with Control Bus Net software package (Wealtec Bioscience Co., Ltd., New Taipei City, Taiwan). Primers were designed and synthesized by Nanjing Genscript Biotech Co., Ltd., (Jianngsu, P. R. China). The results were analyzed using the 2^−ΔΔCt^ method. Sequences of the primers were shown in Table 1.

**Table 1:** Sequences for RT-qPCR primers.

miRNAs	Sequences (5’–3’)
miR-22-3p	F: ACACTCCAGCTGGGAAGCTGC
	R: CTCGCTTCGGCAGCACA
miR-24-3p	F: AGCTTTCAGGCGATCTGGAG
	R: GTCTCAGGCTTGGTCAGTCC
miR-203a-3p	F: CCGGTGAAATGTTTAGGACCACTAG
	R: GCCGCGTGAAATGTTTAGG
miR-320a-3p	F: TATTCGCACTGGATACGACTCCAGC
	R: GTCGTATCCAGTGCAGGGTCCGAGG
miR-320b	F: TCCGAAACGGGAGAGTTGG
	R: GTGCAGGGTCCGAGGT
miR-100-5p	F: ATCATTAAACCCGTAGATCCGAA
	R: AATGGTTGTTCTCCACACTCTCTC
miR-423-5p	F: ATGGTTCGTGGGTGA
	R: GTGCAGGGTCCGAGGT
miR-200b-3p	F: GCGGGCTAATACTGCCTGG
	R: ATCCAGTGCAGGGTCCGAAA
miR-126-3p	F: TATCAGCCAAGAAGGCAGAA
	R: CGTGGCGTCTTCCAGAAT

### Statistical analysis

Each experiment was carried out 3 times. All data were calculated by GraphPad Prism (version 7, GraphPad Software Inc.), and presented as mean ± SD. The Student's *t*-test was used to contrast two groups’ differences, then contrast among multiple groups used the Analysis of Variance (ANOVA) followed by Duncan's post-hoc test. The correlation analysis was performed using Pearson's correlation analysis. The clinical significance of PAF was analyzed in the plasma by the Receiver Operating Curve (ROC) using the Area Under the Curve (AUC); *p* < 0.05 suggested a significant difference.

## Results

### COPD patient characteristics

In this study, a total of 69 individuals including 36 COPD patients as well as 33 healthy volunteers were enrolled. No significant difference shows in sex, age, Body Mass Index (BMI), family history of COPD as well as a history of smoking between the two groups (Table 2).

**Table 2:** Demographic, clinical and biological data of the COPD patients and healthy controls in the miRNA screen study.

Clinicopathologic characteristics	COPD (*n* = 36)	NC (*n* = 33)	*p*-value
Male/Female	19/17	21/12	0.3613
Age (years), mean ± SD	58.56 ± 12.40	63.97 ± 11.76	0.0677
BMI (kg/m^2^), mean ± SD	24.59 ± 3.53	25.92 ± 3.75	0.1351
Family history of COPD			0.2922
No	14	17	
Yes	22	16	
History of smoke			0.4125
No	15	17	
Yes	21	16	
FEV1 (%predicted), mean ± SD	52.81 ± 11.14	94.54 ± 8.55	< 0.0001
FEV1/FVC (%), mean ± SD	59.63 ± 10.32	81.79 ± 6.24	< 0.0001

### mRNA expression profile in COPD patients

**Fig. 1:**
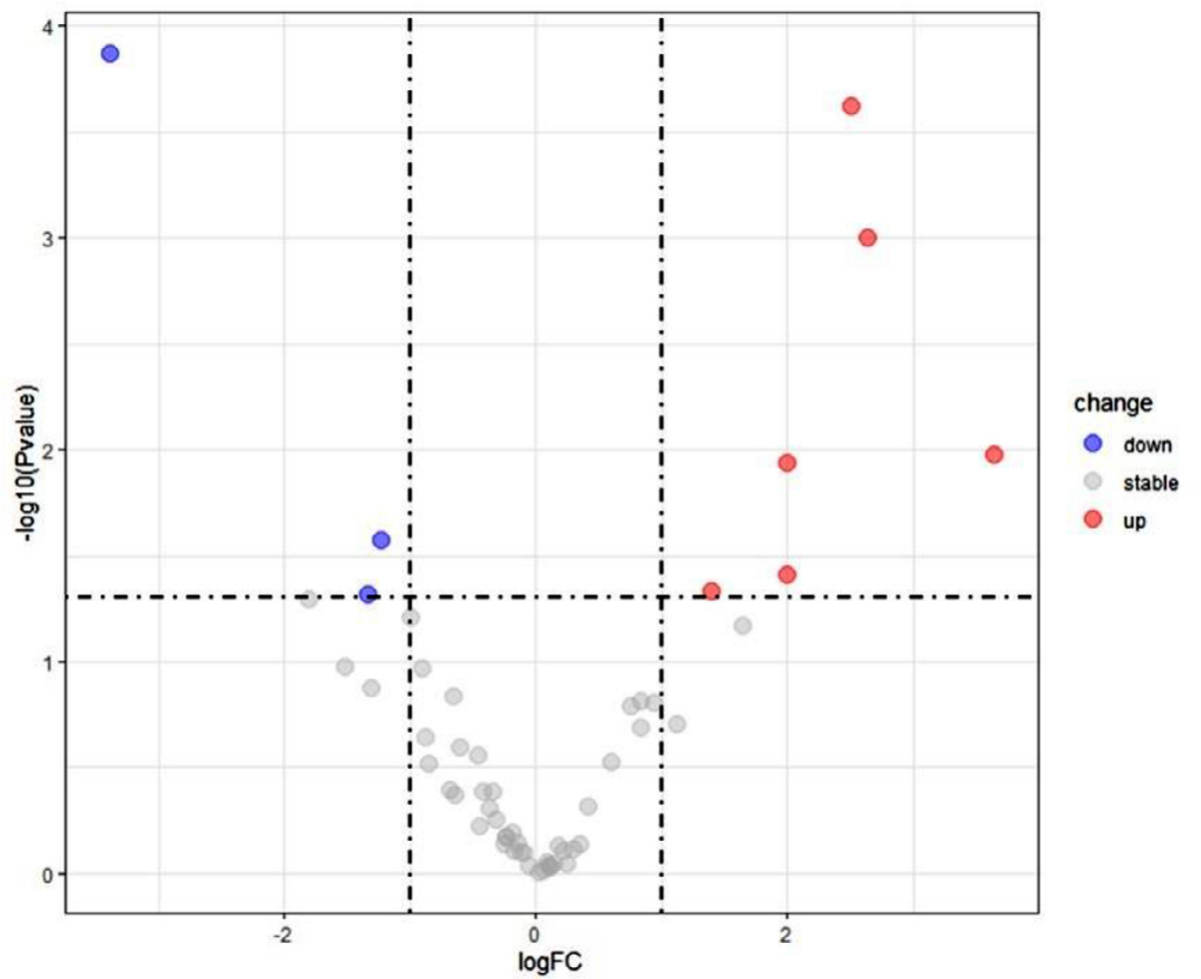
mRNA expression profile in COPD patients. Volcano plots indicated the differentially expressed miRNAs between COPD patients and normal samples.

A total of 55 miRNAs in the plasma of COPD patients were assessed, 6 miRNAs levels were notably increased while 3 miRNAs levels were decreased markedly (Fig. 1). These miRNAs were selected for further analysis.

### Validation of dysregulated miRNAs in COPD patients

**Fig. 2:**
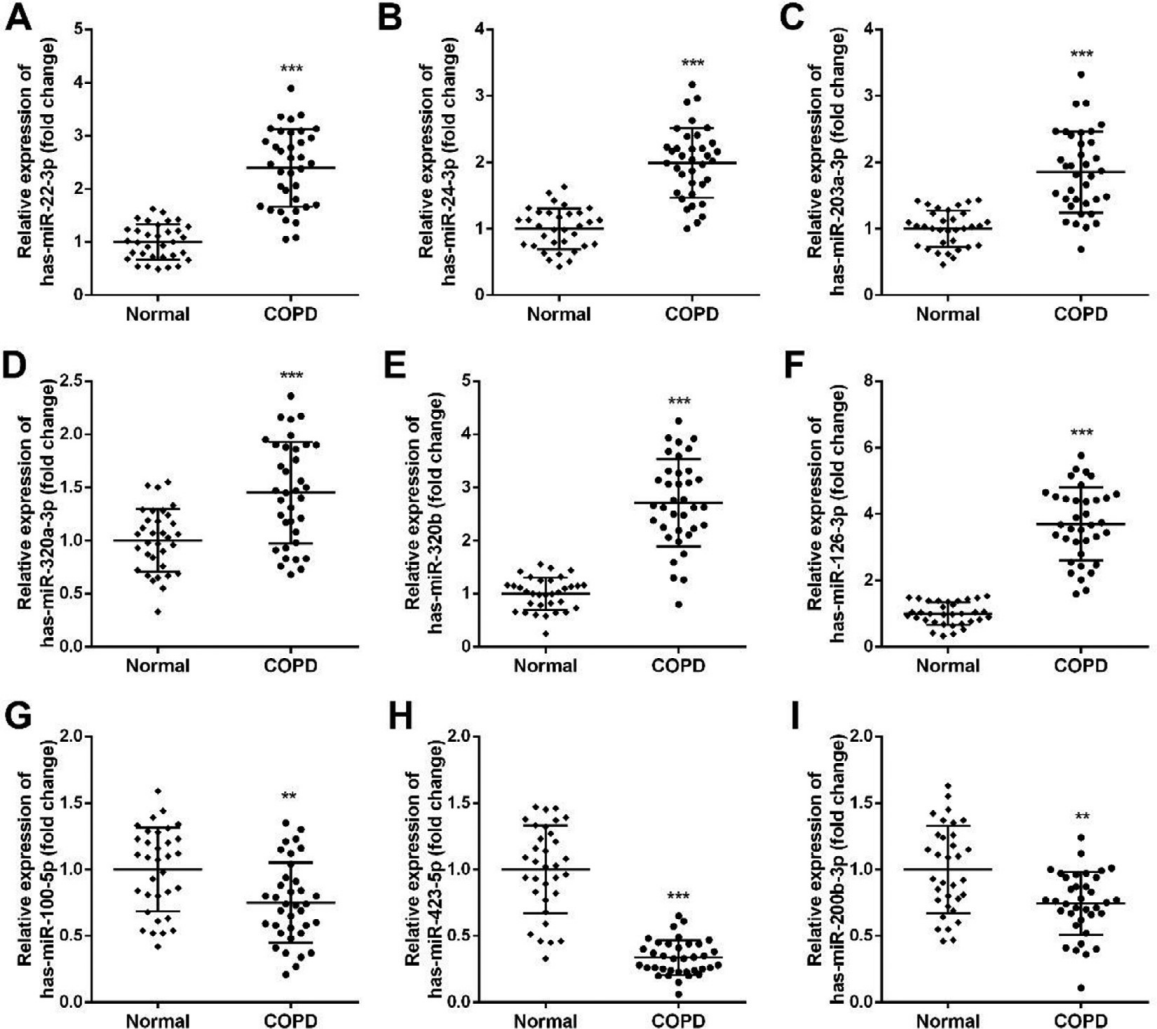
Validation of dysregulated miRNAs in COPD patients. (A‒I) Expression of different miRNAs. ** *p* < 0.01, *** *p* < 0.001 versus normal.

The 9 miRNAs with notably differential expression in COPD patients were verified via RT-qPCR. Compared to the healthy individuals, 6 miRNAs (has-miR-22-3p, has-miR-24-3p, has-miR-203a-3p, has-miR-320a-3p, has-miR-320b, has-miR-126-3p) expression were significant up-regulated (Fig. 2A‒F), nevertheless, 3 miRNAs (has-miR-100-5p, has-miR-423-5p, has-miR-200b-3p) were down-regulated observably (Fig. 2G‒I). Considering that miR-423-5p was notably dysregulated in COPD patients and had not been reported at present, further analysis will focus on miR-423-5p.

### Receiver operating characteristic curves for miR-423-5p

**Fig. 3:**
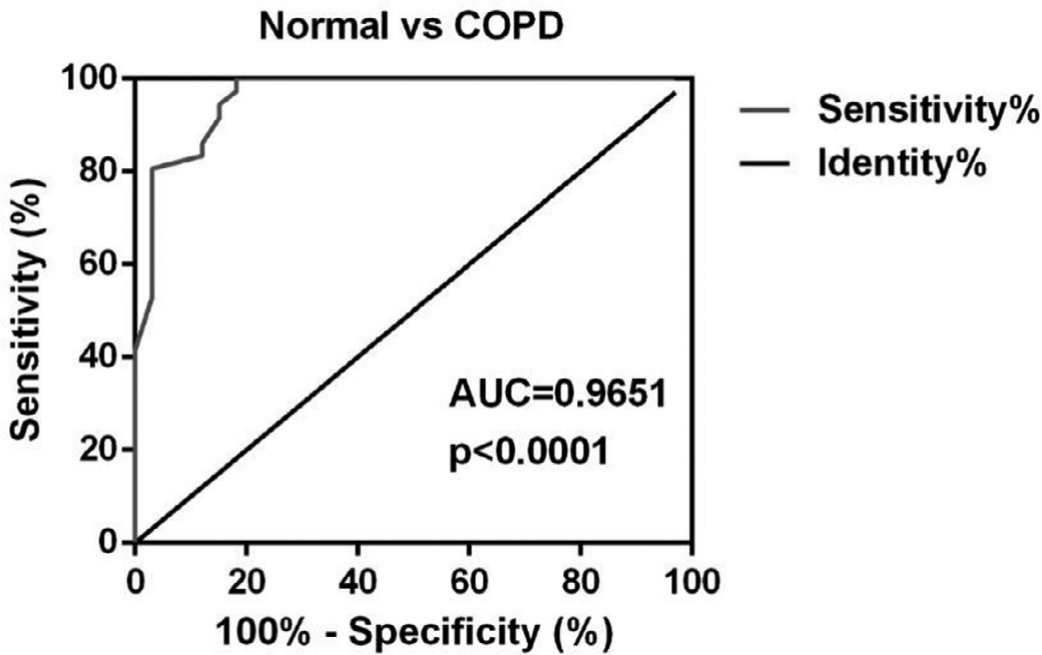
Receiver operating characteristic curves for miR-423-5p. ROC curve analysis of diagnostic efficacy of miR-423-5p.

Compared with the healthy individuals, the receiver operating characteristic curve showed that the AUC of miR-423-5p for the diagnosis of COPD was 0.9651 (95% CI: 0.9269–1) (Fig. 3). Besides, there was no significant difference in sex, age, BMI between miR-423-5p low expression group and high expression group. Besides, more patients with a family history of COPD and smoking longer expressed a low level of miR-423-5p (Table 3).

**Table 3:** Demographic, clinical and biological data of the COPD patients and healthy controls in the miRNA screen study.

Clinicopathologic characteristics	Low (*n* = 25)	High (*n* = 11)	*p*-value
Male/Female	13/12	6/5	0.8879
Age (years), mean ± SD	57.64 ± 11.25	60.64 ± 15.08	0.5121
BMI (kg/m^2^), mean ± SD	24.45 ± 3.30	24.45 ± 4.03	0.3425
Family history of COPD			0.0433*
No	7	7	
Yes	18	4	
History of smoke			0.7598
No	10	5	
Yes	15	6	
Duration of smoking (years)	37.56 ± 4.76	33.00 ± 7.20	0.0346*
FEV1 (%predicted), mean ± SD	51.26 ± 11.48	56.35 ± 9.92	0.2115
FEV1/FVC (%), mean ± SD	58.08 ± 10.78	63.15 ± 8.63	0.1779
FVC (L), mean ± SD	3.57 ± 0.33	3.62 ± 0.22	0.6991

### Relationship between plasma miR-423-5p expression in COPD patients as well as smoking history

**Fig. 4:**
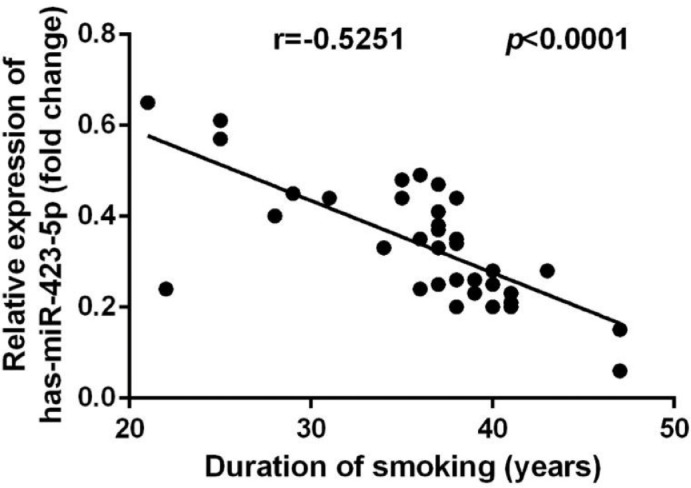
Relationship of plasma miR-423-5p expression in COPD patients as well as smoking history. Correlation between the levels of plasma miR-423-5p as well as the duration of smoking.

The plasma miR-423-5p level in COPD patients was inversely correlated with the duration of smoking (*r* = -0.5251, *p* < 0.0001; Fig. 4).

### Relationship between plasma miR-423-5p expression in the chest tomography results of COPD patients

**Fig. 5:**
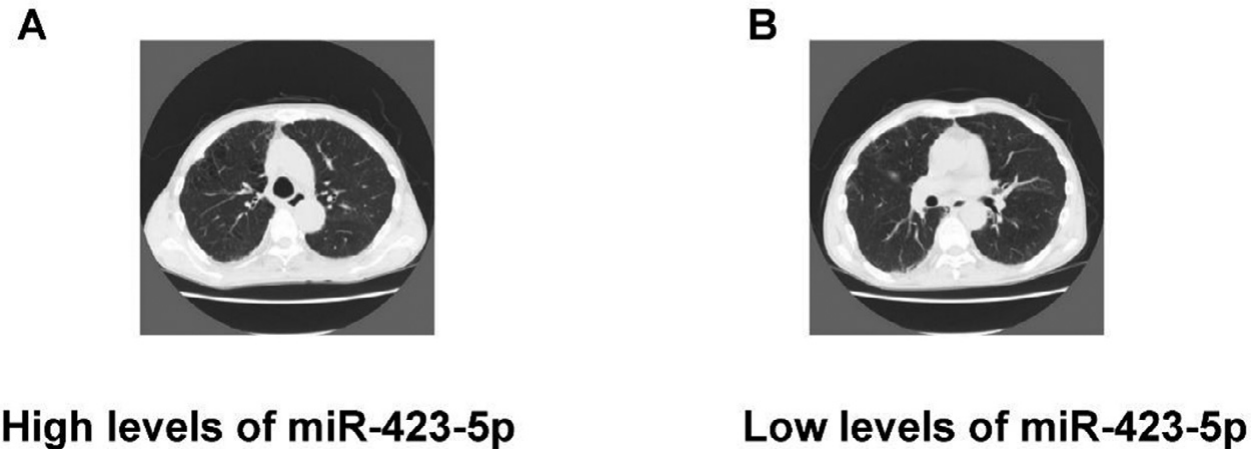
Relationship between plasma miR-423-5p expression and the chest tomography results in COPD patients. The chest tomography results of the COPD patients with the high levels of miR-423-5p (A) and low levels of miR-423-5p (B).

As shown in Fig. 5, the authors found that COPD patients with low levels of miR-423-5p exhibited an obvious disease characteristic compared with COPD patients with high levels of miR-423-5p.

## Discussion

COPD is the third leading cause of death worldwide.^26^ China is a high incidence area of COPD. According to statistics, nearly one-third of the 2.99 billion COPD patients worldwide in 2017 were in China.^27^^,^^28^ In this study, plasma levels of 55 miRNAs were measured from 36 COPD patients as well as 33 healthy individuals, and 9 miRNAs were found to be significantly dysregulated. Among them, 6 miRNAs expression significantly increased while 3 miRNAs were decreased observably. Further analysis of miR-423-5p revealed that the AUC of miR-423-5p for the diagnosis of COPD was 0.9169 (95% CI: 0.8415–0.9923), and its expression level was inversely correlated with the duration of smoking in patients, suggesting that miR-423-5p has potential value as a clinical diagnosis of COPD.

MiRNAs play a vital role in the physiological as well as pathological mechanisms of various respiratory diseases, containing asthma, idiopathic pulmonary fibrosis, bronchiectasis, and COPD.^29^ Lung tissues have been dependent on miRNAs since early embryonic development. The expression of mir-29b in the epithelial cells of COPD patients is significantly decreased.^30^ In addition, miR-335-5p expression was notably decreased in lung fibroblasts of COPD patients.^31^ In this study, 9 miRNAs were significantly dysregulated and RT-qPCR further confirmed that 6 miRNAs (has-miR-22-3p, has-miR-24-3p, has-miR-203a-3p, has-miR-320a-3p, has-miR-320b, has-miR-126-3p) expression were significant up-regulated while 3 miRNAs (has-miR-100-5p, has-miR-423-5p, has-miR-200b-3p) were down-regulated observably.

Reports showed that miR-423-5p takes part in the regulation of the development of various tumors, such as aggravating the development of lung adenocarcinoma by targeting CADM1,^32^ targeting GRIM-19 to promote the progression of prostate cancer,^33^ and targeting STMN1 to inhibit the proliferation and invasion of osteosarcoma.^34^ There is no report of mir-423-5p in COPD. In this study, compared with healthy individuals, the miR-423-5p level in COPD patients was markedly down-regulated, and the AUC for the diagnosis of COPD was 0.9651, indicating that miR-423-5p can be used as a potential diagnostic indicator for COPD. In addition, the miR-423-5p level in patients with COPD was inversely correlated with the duration of smoking (*r* = -0.5251, *p* < 0.0001), and family history also had a notable effect on the decrease of the miR-423-5p level.

In conclusion, the present study's results suggest that 9 miRNAs were dysregulated in COPD patients and miR-423-5p may be a target for COPD diagnosis.

## Authors' contributions

All authors participated in the design, interpretation of the studies and analysis of the data, and review of the manuscript. X Z drafted the work and revised it critically for important intellectual content; Q S, L X and SY S were responsible for the acquisition of the work, Y L and YH W were responsible for the analysis and interpretation of data for the work; MC Z made substantial contributions to the conception or design of the work.

## Funding

This work was supported by Chongqing Science and Health Joint Medical Research Project under Grant no. 2020FYYX229.

## Data availability statement

The datasets used and analyzed during the current study are available from the corresponding author upon reasonable request.

## Declaration of Competing Interest

The authors declare no conflicts of interest.
